# 

**Published:** 1915-03-20

**Authors:** 


### CONTENTS.

PAGE

**Professional Titles, Pleasant and Unpleasant** 545

**Six Months of Hospital War Work** ... ... 546

**Hospital and Institutional News** ... ... ... 547

The D.S.O. for an Army Doctor—Hospital Procedure and Military Patients—Tenders in War-Time—The Regimental Stretcher-Bearers—District Medical Officers and Security of Tenure—The Evil of Annual Appointments—Swansea Hospital and the War—The Teaching of Sex Hygiene—The Migration of Hospitals—New Professor of Pathology at Manchester—The Canadian Emergency Hospital—German Hospital Wards—The Cause and Cure of Perfunctory Fire Drills—Secretary for a Hospital in France—Mr. W. E. Gladstone's Physician—Hospital Servants who Leave without Notice—New Hospital in Glamorganshire—York County Hospital and Soldier Patients—The Pay-Ward Movement in Scotland—Accounts, and Workmen's Representation at Bristol—Cardiff Institute for the Blind—Surgery Lamps and the Lighting Regulations—Rival Claims to a Charitable Bequest—Duty-Free Rectified Spirit in Hospitals?—Bagthorpe Infirmary for Military Patients—New Secretary of the Royal Eye Hospital—This Week's Drug Market.

**The Belgian Field Hospital From Within** ... 553

The Surgeon-in-Chief's Experiences.

**The French Medical Service** ... ... ... 554

The Organisation through French Eyes.

**Soldiers at the London Hospital** ... ... 555

The Method and Procedure Adopted.

**Modern Methods in Rhino-Laryngology** ... 556

Sir St. Clair Thomson's Harveian Lecture.

**A Medical Causerie** ... ... ... ... ... 557

[See over.]

### CONTENTS—(*continued*).

| | PAGE |
|---|---|
| **Round the World** ... ... ... ... ... | 558 |
| Fellow-Workers and the Roll of Honour. | |
| **The Division of In-Patient and Out-Patient Expenditure** ... ... ... ... ... | 559 |
| King Edward's Hospital Fund for London Statistics. | |
| **Gifts and Bequests** ... ... ... ... ... ... | 5[illegible]0 |
| **The Evolution of the Modern Hospital** ... | 561 |
| IV.—Medical Education, Administration, and Finance. | |
| **Legal Notes for Institutional Workers** ... | 562 |
| A Curious Motor-Car Case. | |
| Doctors and the Duplication of Insurance Prescriptions. | |
| **Home-Made Hospital Equipment** ... ... | 563 |
| A Cold-Storage Plant at a Provincial Hospital. | |
| **The War and the Wounded** ... ... ... | 564 |
| Sanitary Features of the Present Campaign. | |
| **Order of St. John of Jerusalem** ... ... | 566 |
| **The First Certified Hospital Fire Brigade** ... | 566 |
| **Medical Appointments** ... ... ... ... | 566 |
| **Doctors' Wills** ... ... ... ... ... ... | 566 |
| **Buildings Contemplated** ... ... ... ... | 566 |
| **The Book Market** ... ... ... ... ... | 566 |
| **The Institutional Worker** ... ... (Suppl.) | 1 |
| Institutional Workers and their Work : II. The Assistant Secretary. | |
| Question for March. | |
| Questions Invited. | |
| Employment and Training. | |
| "The Hospital Secretary." | |

